# Effect of Tumor Regression Grade on Survival and Disease-Free Interval in Patients Operated on for Locally Advanced Rectal Cancer

**DOI:** 10.3390/cancers16101797

**Published:** 2024-05-08

**Authors:** Fernando Mendoza-Moreno, Manuel Díez-Alonso, Belén Matías-García, Enrique Ovejero-Merino, Cristina Vera-Mansilla, Ana Quiroga-Valcárcel, Alma Blázquez-Martín, Rubén Jiménez-Martín, Inmaculada Lasa-Unzúe, Miguel A. Ortega, Melchor Alvarez-Mon, Alberto Gutiérrez-Calvo

**Affiliations:** 1 Department of General and Digestive Surgery, Príncipe de Asturias Teaching Hospital, 28805 Alcala de Henares, Spain; manuelmariano.diez@salud.madrid.org (M.D.-A.); belenmg3@gmail.com (B.M.-G.); enrique.ovejero@gmail.com (E.O.-M.); cvera@salud.madrid.org (C.V.-M.); aquirogavalcarcel@gmail.com (A.Q.-V.); ablazquezmartin@gmail.com (A.B.-M.); ruben.jimenez@salud.madrid.org (R.J.-M.); ilasau@gmail.com (I.L.-U.); agutierrezcalvo@telefonica.net (A.G.-C.); 2 Department of Medicine and Medical Specialities, Faculty of Medicine and Health Sciences, University of Alcalá, 28801 Alcalá de Henares, Spain; miguel.angel.ortega92@gmail.com (M.A.O.); mademons@gmail.com (M.A.-M.); 3 Ramón y Cajal Institute of Sanitary Research (IRYCIS), 28034 Madrid, Spain; 4 Immune System Diseases-Rheumatology and Internal Medicine Service, University Hospital Príncipe de Asturias, Network Biomedical Research Center for Liver and Digestive Diseases (CIBEREHD), 28806 Alcalá de Henares, Spain

**Keywords:** rectal cancer, colorectal carcinoma, complete pathological response, staging, chemotherapy, radiation therapy

## Abstract

**Simple Summary:**

The neoadjuvant treatment consisting of chemoradiotherapy has shown significant improvement in survival and recurrence rate in patients undergoing rectal cancer surgery. In recent years, different neoadjuvant regimens have been proposed as initial therapy in patients with locally advanced rectal cancer. However, the response to neoadjuvant treatment (understood as the degree of tumor regression) is not the same in all patients. The aim of our study has been to analyze the different factors (both preoperative and postoperative) that influence a better degree of tumor regression, and therefore a greater survival and disease-free interval.

**Abstract:**

Introduction: Colorectal cancer is the fourth leading cause of cancer-related death in both men and women in our population. In this regard, rectal cancer accounts for more than half of colorectal cancer deaths, and its incidence is expected to increase in the coming years. There have been significant changes in neoadjuvant therapy regimens, with promising results, as demonstrated by the recent RAPIDO and PRODIGE23 studies. Around 40% of patients diagnosed with locally advanced rectal cancer show some degree of response to neoadjuvant treatment, with complete tumor regression observed in up to one in five patients. Materials and Methods: Retrospective observational study. A total of 181 patients with locally advanced rectal cancer treated with neoadjuvant chemoradiotherapy followed by surgery were analyzed. Clinical and pathological data were collected from the patients, including assessment of tumor regression through histopathological studies after surgery. The Mandard tumor regression grading system was used to categorize tumor response into different grades. Results: The results showed a significant association between the degree of tumor regression and several important clinical outcomes. Specifically, patients with higher tumor regression had significantly better disease-free survival than those with less regression (*p* = 0.004). In addition, tumor regression was also correlated with the incidence of local recurrence (*p* = 0.018) and distant metastasis (*p* = 0.032). These associations suggest that tumor responsiveness to neoadjuvant therapy may influence the long-term progression of the disease. Regarding tumor deposits and the presence of lymphadenopathy, these factors were also found to be significantly associated with clinical outcomes. Patients with tumor deposits had a higher incidence of local recurrence (*p* = 0.025) and distant metastases (*p* = 0.041), while the presence of lymphadenopathy increased the risk of local recurrence (*p* = 0.013). These findings highlight the importance of evaluating not only tumor regression but also other pathological markers to predict prognosis and guide clinical management. Conclusions: The degree of tumor regression was not an independent predictor of survival compared to other variables such as nodal stage and presence of tumor deposits. This indicates that while tumor regression is an important factor, other elements also play a crucial role in determining the prognosis of patients with locally advanced rectal cancer. This study provides additional evidence for the importance of tumor regression, tumor deposits, and lymphadenopathy as predictors of clinical outcomes in patients with rectal cancer treated with neoadjuvant chemoradiotherapy.

## 1. Introduction

Colorectal cancer is the fourth leading cause of cancer-related death in both men and women in our population [1]. Thus, rectal cancer accounts for more than half of colorectal cancer deaths, and its incidence is expected to increase in the coming years [2,3,4]. However, its prognosis in recent decades in terms of survival, locoregional recurrence, and development of distant metastases has improved significantly with the incorporation of neoadjuvant treatment consisting of chemoradiotherapy (CRT) and surgery (resection + mesorectal excision).

In recent years, there have been significant changes in neoadjuvant therapy regimens, with promising results, as demonstrated by the recent RAPIDO and PRODIGE23 studies. Around 40% of patients diagnosed with locally advanced rectal cancer show some degree of response to neoadjuvant treatment, with complete tumor regression observed in up to one in five patients [1].

However, there are still unclear aspects regarding neoadjuvant CRT. It is not known why some patients show a higher degree of tumor regression after neoadjuvant treatment. In addition, the significance of tumor regression on the long-term evolution of the disease is unclear.

In this study, we aimed to analyze the relationship between the degree of tumor regression after neoadjuvant CRT and the postoperative evolution of patients. Our working hypothesis was that patients in whom a complete or near-complete pathological response is detected show better disease progression parameters than those in whom a poor or null response is observed.

## 2. Materials and Methods

Retrospective Cohort Study: This is a retrospective observational study on 181 patients diagnosed with locally advanced rectal adenocarcinoma treated between June 2009 and May 2021. The patients were selected from the data collected in the computerized file of the Coloproctology Unit, filled in prospectively during these years. The study was approved by the Ethics Committee of the Príncipe de Asturias Teaching Hospital (Reference OE61/2023).

The objective of our study was to analyze the relationship between the degree of tumor regression after neoadjuvant CRT and overall survival (OS), disease-free interval (DFS), locoregional recurrence (LR-FS), and metastasis (M-FS) and to compare the results obtained in patients with a complete or near-complete pathological response (Mandard grades 1 and 2) versus those with a poor or null response (Mandard grades 3, 4, and 5). We also aimed to analyze the risk of recurrence and death associated with the degree of tumor regression.

Inclusion and Exclusion Criteria: Patients were included based on confirmed histopathology of adenocarcinoma NOS (carcinoma with glandular and ductal differentiation that does not present histomorphological characteristics of another carcinoma), age over 18 years, preoperative local staging with pelvic magnetic resonance imaging (MRI), preoperative stage II/III, tumor located in the middle (9 to 7 cm) or lower (within 6 cm of the anal verge) rectum, neoadjuvant treatment consisting of chemoradiotherapy, and subsequent curative surgical resection.

Exclusion criteria included tumor location in the upper rectum, presence of synchronous tumor, presence of distant metastases, TNM stage I, failure to complete neoadjuvant treatment, administration of short-course radiotherapy, or patients who were not ultimately surgically intervened.

Treatment: After undergoing a colonoscopy with biopsy confirming adenocarcinoma of the rectal lesion, a thoracoabdominopelvic computed tomography (CT), a pelvic magnetic resonance imaging (MRI), and blood tests including tumor marker determination were performed, and all patients were presented to the Digestive Tumors Committee. A multidisciplinary decision was made to initiate adjuvant treatment in patients diagnosed with locally advanced rectal adenocarcinoma with planned subsequent surgical intervention. Neoadjuvant treatment consisted of radiotherapy (50.4 Gy administered in 1.8 Gy/fraction daily) administered in 25 sessions over 5 weeks. Capecitabine was used as a concomitant chemotherapeutic agent at a dose of 850 mg/m^2^, twice daily. After a minimum period of 6 weeks (interval 6–12 weeks), patients underwent surgical intervention. After surgery and depending on the histopathological result of the surgical specimen, patients received adjuvant chemotherapy 4 weeks after surgery, consisting of four to six cycles of XELOX (Oxaliplatin and Capecitabine) administered every 28 days.

The degree of tumor regression was studied in the resection specimen and classified anatomopathologically according to the Mandard classification. For this study, patients were classified into two groups: (A) good pathological response, which included Mandard grades 1 (absence of residual tumor cells and fibrosis in the rectal wall) and 2 (sparse nests of residual tumor cells within the fibrosis), and (B) poor pathological response, which included Mandard grades 3, 4, and 5 (predominant fibrosis over residual tumor nests, predominant residual tumor nests over fibrosis, or absence of regressive changes).

Statistical Analysis: Data on demographic, analytical, tumor-related, and treatment-related variables were collected. The variables were collected in a Microsoft Excel 2019 spreadsheet. Statistical analysis was performed using SPSS software (v.23) (IBM, Armonk, NY, USA). Preoperative variables (known before surgical intervention) included the interval between completion of neoadjuvant treatment and surgery, tumor location (middle or lower rectum), clinical stage, gender, CEA, CA19.9, hemoglobin, and albumin. Neutrophil/lymphocyte and neutrophil/leukocyte ratios were calculated based on preoperative blood tests. Postoperative variables (known only after surgical intervention) included TNM tumor stage, degree of regression according to the Mandard scale, circumferential margin involvement, tumor differentiation grade, lymphovascular invasion, presence of tumor deposits, or subsequent systemic treatment with chemotherapy.

The distribution of patient and tumor characteristics between groups of tumor regression grade was compared using the chi-squared test. Survival time in the cohort of rectal cancer patients was described, with follow-up defined as the time between surgery and death or the last medical appointment. Survival at 3 and 5 years after diagnosis and median survival were estimated using the Kaplan–Meier estimator with 95% confidence intervals. Overall survival (OS) in this series is equivalent to cancer-related survival, as all deaths were caused by rectal cancer.

Next, the study focused on the association between tumor regression grade after CRT and patient survival and recurrence. Finally, the effect of tumor regression grade on clinical outcome endpoints was evaluated using Cox proportional hazard regression after adjusting for the effect of confounding variables. A *p*-value <0.05 was considered statistically significant.

## 3. Results

**Figure 1:**
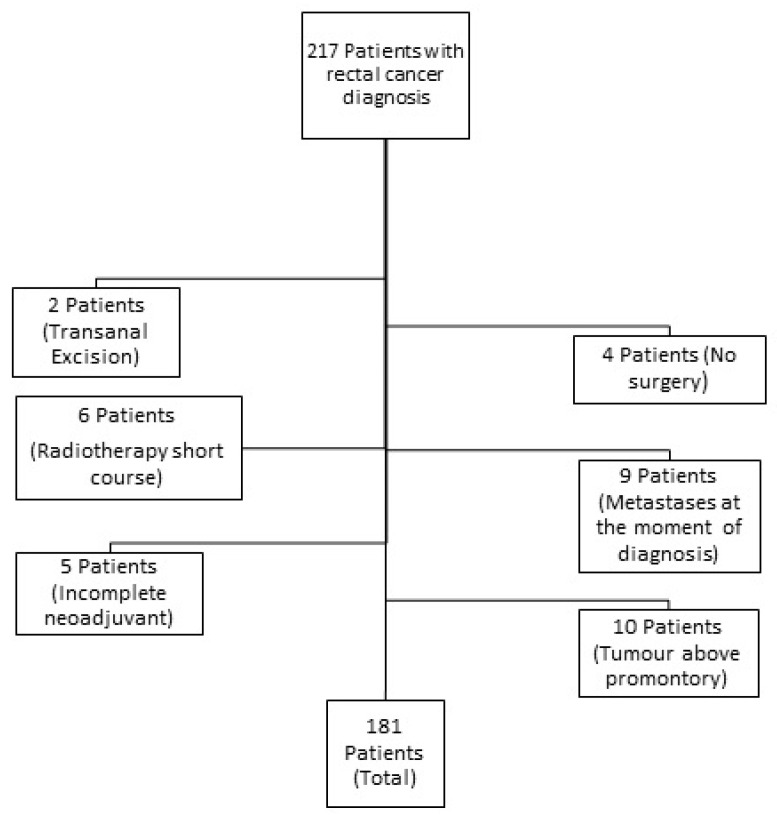
Flowchart detailing the selection of the patients in this study.

During the described period, 492 patients diagnosed with malignant rectal neoplasia underwent surgery in our center. After considering the exclusion criteria, 181 patients, 119 (65.7%) males and 62 (34.3%) females, were included in the analysis (Figure 1). The mean age was 62 ± 11 years. The clinical–pathological characteristics are shown in Supplementary Table S1.

The mean follow-up was 98 ± 54 months (median 90 months). The most frequent tumor location was the lower rectum (97 patients, 53.6%). According to pre-treatment staging using pelvic MRI, 28 patients (15.5%) were classified as TNM stage II and 153 (84.5%) as stage III.

The interval between completion of neoadjuvant treatment and surgery was less than 8 weeks in 87 (48.1%) patients, while 94 (51.9%) were operated on between 8 and 10 weeks later. In our results, the statistical significance of the effect of the interval from completion of neoadjuvant treatment (chemoradiotherapy) to surgical intervention turned out to be non-significant (*p* = 0.314) (Table 2).

Pathological analysis of the resected tumors showed lymph node involvement in 50 patients (27.6%): 39 (21.5%) with pN1 and 11 (6.1%) with pN2. The number of isolated lymph nodes was 8 ± 6 (median 7), while the number of positive lymph nodes was 0.7 ± 1.9 (median 0).

The tumor regression observed, according to the Mandard classification, was as follows: 32 patients (17.6%) with Mandard grade 1, 69 patients (38.1%) with Mandard grade 2, 67 patients (37%) with Mandard grade 3, and 13 patients (7.2%) with Mandard grade 4. Accordingly, 101 patients (55.8%) exhibited a good pathological response, while 80 patients (44.2%) showed a poor response.

Tumors with a poor histopathological response to neoadjuvant CRT showed a higher frequency of nodal ypN2 involvement (81.8% vs. 18.2%) (*p* = 0.001), tumor deposits (87.5% vs. 12.5%) (*p* = 0.001), perineural invasion (82.4% vs. 17.6%) (*p* = 0.001), vascular invasion (87.5% vs. 12.5%) (*p* = 0.001), poor differentiation grade (76.9% vs. 32.1%) (*p* = 0.019), and circumferential margin involvement (90.1% vs. 9.1%) (*p* = 0.003) (Supplementary Table S1). Preoperative radiological staging and the interval between neoadjuvant CRT and surgery showed no relationship with the degree of tumor regression, as the response grade was similar in all defined categories of these variables.

### 3.1. Recurrence

In this series of patients, 41 (22.6%) experienced some form of recurrence. Of these, 38 patients (92.7%) had a distant recurrence, while 5 patients (7.3%) had a locoregional recurrence. The most common site of distant tumor dissemination was the lung (46.3%), followed by the liver (21.9%). Only two patients had both locoregional and distant recurrence (4.9%). Disease-free survival (DFS), including all types of recurrence, according to the Kaplan–Meier method, was 95%, 82%, and 76% at 12, 36, and 60 months, respectively.

**Figure 2:**
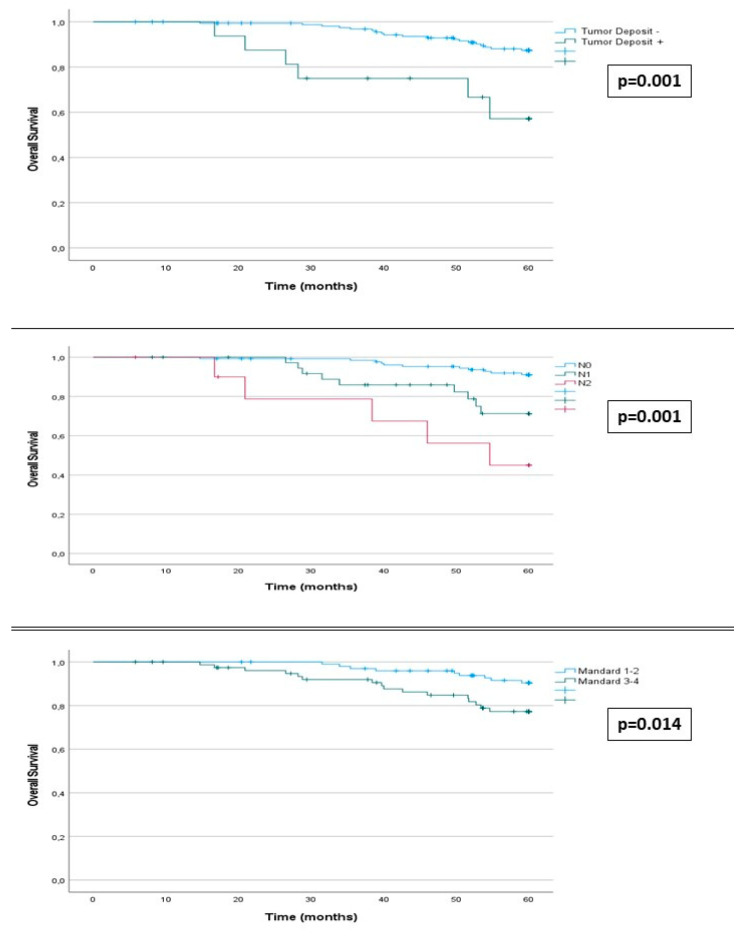
Kaplan–Meier estimates. Horizontal bar denotes median survival.

Among patients with a good pathological response, 15 (14.8%) experienced recurrence, compared to 26 (32.5%) in those with a poor response (*p* = 0.004). DFS at 60 months post-surgery was related to the degree of tumor regression, being higher in tumors with greater regression (87% in grade 1, 83% in grade 2, 66% in grade 3, and 51% in grade 4) (*p* = 0.011). DFS was higher in patients with a good response (84%) compared to those with a poor response (64%) (*p* = 0.002) (Supplementary Table S2) (Figure 2).

Among other postoperative variables related to the tumor, the results of stage ypN were noteworthy. Lower DFS was observed in patients with more advanced ypN stage (85% in N0, 54% in N1, 34% in N2) (*p* < 0.001). Recurrence occurred in 19 (18%) patients in stage N0, 16 (41%) in N1, and 6 in N2 (54%) (*p* < 0.001). The presence of tumor deposits was also associated with lower DFS (36% vs. 80%) (*p* < 0.001). Recurrence occurred in 31 (18%) patients with tumors without tumor deposits and 10 (62.5%) with deposits (*p* < 0.001) (Supplementary Table S3) (Figure 2).

Among preoperative variables, only NLS > 5 showed a statistically significant relationship with DFS (54% vs. 78%) (*p* = 0.001) (Table 1). Among other postoperative variables, a relationship was observed between DFS and ypT stage (82% in T0, 95% in T1, 86% in T2, 62% in T3, and 40% in T4) (*p* < 0.001), circumferential margin involvement (37 vs. 78%) (*p* < 0.001), lymphovascular invasion (31% vs. 8%) (*p* < 0.001), perineural infiltration (31% vs. 80%) (*p* < 0.001), and tumor differentiation grade (Table S3).

In multivariate analysis, the risk of tumor recurrence associated with the degree of tumor regression did not reach statistical significance (Table 3). The factors that showed independent predictive value were ypN stage (N1: HR, 4.126; 95% CI: 1.514–11.247; N2: HR, 2.126; 95% CI: 1.148–5.383) and the presence of tumor deposits (HR: 3.067; 95% CI: 1.347–6.981) (Table 1).

**Table 1:** Predictive factors of disease-free survival analyzed using Cox’s proportional hazards model.

	*p* Value	HR	95% CI
ypN STAGE N0 N1 N2	*p* = 0.009	1 4.126 2.126	1.514–11.247 1.148–5.383
TUMOR DEPOSIT Absent Present	*p* = 0.008	1 3.067	1.347–6.981
MANDARD TUMOR REGRESSION GRADE 1–2 3–4–5	0.217	1 1.659	0.742–3.707

HR: hazard ratio; 95% CI: 95% confidence interval.

### 3.2. Locoregional Recurrence

The LR-FS in the entire series was 98%, 97%, and 96% at 12, 36, and 60 months, respectively. LR-FS was significantly higher among patients whose tumors showed a good response compared to those with a poor response (99% vs. 91%) (*p* = 0.032). There was one case (0.9%) of local recurrence among patients with a complete pathological response and four (5%) among those with a poor response (*p* = 0.172).

Among the other post-surgical variables, only a relationship between LR-FS and the presence of tumor deposits was observed (Table 2). LR-FS was lower among patients in whom tumor deposits were detected in the specimen (76% vs. 97%) (*p* = 0.01).

**Table 2:** Predictive factors of metastasis-free survival analyzed using Cox’s proportional hazards model.

	*p* Value	HR	95% CI
ypN STAGE N0 N1 N2	0.020	1 4.223 2.503	1.316–13.547 1.049–5.972
TUMOR DEPOSIT Absent Present	0.031	1 2.670	1.096–6.509
MANDARD TUMOR REGRESSION GRADE 1–2 3–4–5	0.545	1 1.294	0.562–2.978

HR: hazard ratio; 95% CI: 95% confidence interval.

### 3.3. Distant Recurrence

M-FS in the entire series was 95%, 85%, and 77% at 12, 36, and 60 months, respectively. Patients with a good tumor response had a higher M-FS than those with a poor response (85% vs. 67%) (*p* = 0.003). A total of 14 (13.8%) patients with a good response experienced metastases compared to 24 (30%) with a poor response (*p* = 0.004).

Among the other post-surgical variables (Table 2), M-FS was related to the ypN stage (87% in N0, 54% in N1, and 34% in N2) (*p* < 0.001). A total of 16 patients (12.2%) in stage N0, 16 (41%) in N1, and 6 (54.5%) in N2 experienced metastases (*p* < 0.001). Likewise, the presence of tumor deposits was associated with lower M-FS (36% vs. 81%) (*p* < 0.001): 28 (16.9%) patients without tumor deposits experienced metastases compared to 10 (62.5%) with tumor deposits (*p* < 0.001).

Among pre-surgical variables, only tumor location was associated with lower M-FS (70% in tumors located in the upper rectum vs. 83% in lower rectum tumors) (*p* = 0.05) (Table 1). Among the post-surgical variables (Table 2), M-FS was also related to the ypT stage (85% in T0, 95% in T1, 86% in T2, 65% in T3, and 40% in T4) (*p* = 0.001), circumferential margin involvement (37% vs. 80%) (*p* < 0.001), presence of vascular invasion (31% vs. 82%) (*p* < 0.001), and perineural invasion (31% vs. 82%) (*p* < 0.001).

In multivariate analysis, the risk of developing metastases associated with the degree of tumor regression did not reach statistical significance (Table 2). The factors that showed independent predictive value were ypN stage (N1, HR: 4.223; 95% CI: 1.316–13.547) (N2, HR: 2.503; 95% CI: 1.049–5.972) and the presence of tumor deposits (HR: 2.067; 95% CI: 1.096–6.509).

### 3.4. Survival

The OS in our series, according to the Kaplan–Meier method, was 97%, 90%, and 84% at 12, 36, and 60 months, respectively. Survival was higher in patients with tumors showing a higher degree of tumor regression. In patients with a good response, survival was 90% compared to 77% in patients with a poor response (*p* = 0.014). A total of 9 patients (8.9%) with a good tumor response died compared to 16 (20%) with a poor response (*p* = 0.027).

**Figure 3:**
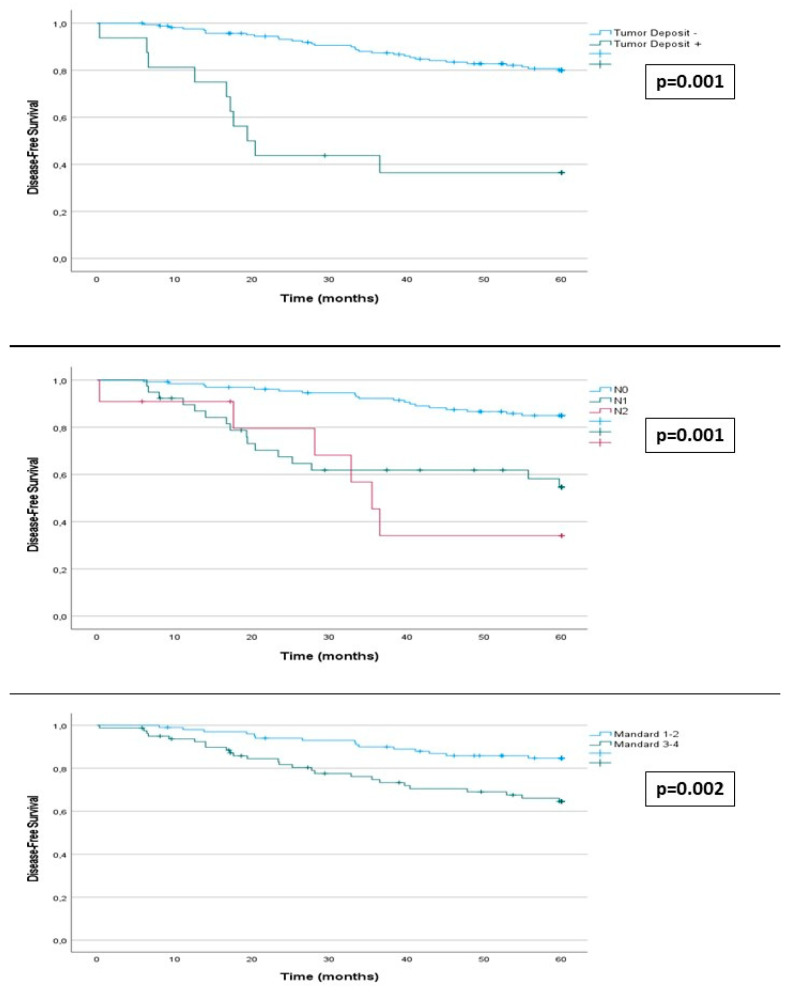
Kaplan–Meier estimates. Horizontal bar denotes median survival.

Among the other post-surgical variables (Table 2), OS was related to the ypN stage (91% in N0, 71% in N1, and 45% in N2) (*p* = 0.001). A total of 11 (8.4%) patients with N0 tumors died, compared to 9 (23%) with N1 tumors and 5 (45.5%) with N2 tumors (*p* < 0.001). Also, the presence of tumor deposits was associated with lower OS (57% vs. 87%) (*p* = 0.001); 19 (11.5%) patients without tumor deposits died compared to 6 (37.5%) with deposits (*p* = 0.012) (Supplementary Table S2) (Figure 3).

Among the other pre-surgical variables, only the location of the primary tumor showed a statistically significant relationship with OS (90% in rectum among patients with lower rectum tumors vs. 76% in middle rectum tumors) (*p* = 0.032) (Table 1). Among the post-surgical variables (Table 2), OS was lower in patients with more advanced stages of ypT stage (85% in T0, 95% in T1, 93% in T2, 79% in T3, and 40% in T4) (*p* = 0.002), circumferential margin involvement (50% vs. 84%) (*p* < 0.001), tumors with poor tumor differentiation (67% vs. 86%) (*p* = 0.046), presence of vascular infiltration (65% vs. 86%) (*p* = 0.009), and perineural invasion (64% vs. 86%) (*p* = 0.004). Postoperative adjuvant chemotherapy was not associated with better postoperative survival (81% vs. 92%) (*p* = 0.076) (Supplementary Table S3) (Figure 3).

In the multivariate analysis, the risk of death associated with the degree of tumor regression did not reach statistical significance (Table 3). The factors that showed an independent predictive value on OS were ypN stage (N1, HR: 3.714; 95% CI: 1.361–10.136) (N2, HR: 2.236; 95% CI: 0.989–5.056), presence of tumor deposits (HR: 2.555; 95% CI: 1.067–6.118), vascular infiltration (HR: 3.157; 95% CI: 1.474–6.761), and localization of primary tumor (HR: 0.512; 95% CI: 0.270–0.969).

**Table 3:** Predictive factors of overall survival analyzed using Cox’s proportional hazards model.

	*p* Value	HR	95% CI
LOCALIZATION OF PRIMARY TUMOR Medium Low	0.040	1 0.512	0.270–0.969
ypN STAGE N0 N1 N2	0.024	1 3.714 2.236	1.361–10.136 0.989–5.056
TUMOR DEPOSIT Absent Present	0.035	1 2.555	1.067–6.118
VASCULAR INFILTRATION Absent Present	0.003	1 3.157	1.474–6.761
MANDARD TUMOR REGRESSION GRADE 1–2 3–4–5	0.228	1 1.538	0.764–3.097

HR: hazard ratio; 95% CI: 95% confidence interval.

## 4. Discussion

Neoadjuvant treatment consisting of preoperative radiotherapy (RT) combined with surgery (total mesorectal excision) has improved the survival and recurrence rates of patients diagnosed with rectal cancer [5]. It is well known that the prognosis of rectal cancer is linked to the development of locoregional recurrence and distant metastases. While Stage I generally does not require neoadjuvant treatment due to low incidence of recurrence and good survival, locally advanced stages (stages II and III) require adjuvant treatment as local recurrence varies between 6 and 33% and survival rates are around 61.6–70% [6].

In our series, we found a complete tumor response after neoadjuvant radiotherapy in 32 (17.6%) patients and an almost complete response in 69 (38.1%). We observed that these patients had higher recurrence-free survival, both overall (84% vs. 64%; *p* = 0.002) and in the form of locoregional recurrence (99% vs. 91%; *p* = 0.032) or metastasis (85% vs. 67%; *p* = 0.003), as well as a longer overall survival (90% vs. 77%; 0.014), than patients with a poor histopathological response.

The first system to assess the degree of tumor regression was described by Mandard in 1994 to evaluate the response to preoperative chemotherapy in esophageal cancer patients [7]. Since then, several classification systems have been proposed (Dwoak, AJCC, Ryan), although there is no consensus on the superiority of any of them. There are several systems for classifying the degree of tumor regression after neoadjuvant treatment (Mandard, Dworak, AJCC, MSKCC, or Rodell). In our series, the Mandard classification was chosen because some patient records were from 2009. Furthermore, this scale is considered more precise as it provides five levels of classification of tumor regression grade response. However, for authors like Trakarnsanga et al., five-tier systems do not show a clear advantage over three-tier systems. Among three-tier systems, the MSKCC scheme is more accurate in predicting recurrence compared to Mandard and Dowrak/Rödel systems, as measured by concordance. The concordance index of the four-tier AJCC system is slightly higher than that of the three-tier MSKCC system, which may result from the additional category introduced in the AJCC system [8].

Neoadjuvant RT reduces tumor size and improves its resectability. Additionally, it has effects on tumor histopathology. In approximately 60–80% of patients who have received neoadjuvant treatment, a decrease or regression in the anatomical extent of the tumor classified according to the TNM system (downstaging) is observed. In addition, a complete tumor response is detected in 12% of patients treated with conventional RT and up to 20–25% in those who have received oral neoadjuvant treatment [9] (Papaccio et al.). In our work, we recorded a complete tumor response in 17.6% of patients, which is in agreement with published findings.

The degree of tumor response to neoadjuvant RT is known to be related to tumor progression. Several publications have reported that patients with a higher degree of response had higher recurrence-free survival, both overall and in the form of locoregional or metastatic recurrence, and longer survival. However, the prognostic value of the regression grade in predicting the course of the disease compared to other coincident variables is not clear.

We found in our work that the prognosis associated with the degree of tumor regression did not reach the level of statistical significance with respect to the risk of recurrence or death in multiple regression analyses, taking into account the weight of other coincident variables. The degree of regression did not show independent predictive value. That is, although the degree of tumor regression was related to the evolution of tumor disease, the evolutionary prognosis depended more on the ypN stage and the presence of tumor deposit. In the multiple regression analysis, ypN stage showed an independent predictive value on disease-free survival (N1: HR, 4.126; N2: HR, 2.126), metastasis-free survival (N1, HR, 4.223; N2, HR: 2.503), and overall survival (N1, HR, 3.714; N2, HR: 2.236). Tumor deposits showed an independent predictive value on disease-free survival (HR: 3.067; 95% CI: 1.347–6.981), metastasis-free survival (HR: 2.067; 95% CI: 1.096–6.509), and overall survival (HR: 2.555; 95% CI: 1.067–6.118).

To interpret these findings, it is necessary to consider that 81.8% of the tumors with a poor histopathological response observed in our study presented ypN2 lymph node involvement, and 87.5% had tumor deposits (*p* = 0.001). Perineural invasion (82.4% vs. 17.6%) (*p* = 0.001), vascular invasion (87.5% vs. 12.5%) (*p* = 0.001), poor differentiation grade (76.9% vs. 32.1%) (*p* = 0.019), and circumferential margin involvement (90.1% vs. 9.1%) (*p* = 0.003) were also more frequent in this group of tumors. These are all factors whose association with poor prognosis is established. Furthermore, the cumulative survival curve graphs show a much more marked separation in ypN and tumor deposits than in the degree of histopathological response. We believe that this indicates that when determining the course of patients, it seems less important whether the resected tumor shows signs of histopathological response to RT; what determines evolution is whether the tumor persists with negative histopathological prognostic features, especially positive ypN and tumor deposits.

It is possible, as suggested by Kasheri et al., that the degree of regression is a histopathological marker of sensitivity to radiotherapy and chemotherapy rather than a factor associated with evolutionary prognosis. Tumors showing a high degree of response may represent a biologically selected group with greater sensitivity to chemoradiotherapy [10]. By extension, and as a mere hypothesis, we might think that this increased sensitivity could justify the administration of postoperative adjuvant chemotherapy, but this would require prospective and randomized studies. In our patients, we indicated the administration of postoperative adjuvant chemotherapy based on the findings in the resected tumor specimen, and in this analysis, we did not detect that postoperative adjuvant treatment was associated with higher disease-free survival or overall survival. We are aware that this was a retrospective study and not specifically designed for this type of analysis, so the value of this finding is limited.

The ypN stage and tumor deposits are two recognized prognostic factors in CRC. It is well known that metastatic lymph node involvement decreases the survival of these patients. Authors such as Benedek et al. have described survival rates of 75% for ypN0 patients, 47.1% for ypN1, and 50% for ypN2 at 5 years [6]. We have observed this relationship in our series, showing a higher number of relapses and lower survival in patients with greater lymph node involvement.

While the origin of tumor deposits is not clear, several hypotheses establish their origin as residual tumors after primary regression, while other authors suggest they originate from metastatic lymph nodes with perineural or lymphovascular involvement [11]. Either way, the prevalence of tumor deposits in patients with rectal cancer ranges from 10.7% to 34.8% [6,12]. In our study, we observed a lower prevalence, which was 8.28%. Several authors have described a significant decrease in the survival of these patients (from 80.7% to 68.8%) in the presence of tumor deposits, as well as a higher risk of locoregional recurrence (from 2.7% to 6.3%) and distant metastasis (from 14.3% to 38.9%) [13]. This can also be observed in our series, both for lymph node involvement and for the presence of tumor deposits.

In recent years, in relation to new neoadjuvant treatment regimens, the influence of various factors, both preoperative and postoperative, on the degree of tumor regression has been analyzed [14]. One of them has been the determination of the interval between the completion of neoadjuvant treatment and surgery. In our series, we did not observe significant differences in patients operated on before 8 weeks compared to those operated on at a longer interval in terms of survival or disease-free interval. Published studies such as GRECCAR-6, where two types of intervals are analyzed (7 versus 11 weeks), conclude that there are no significant differences in terms of complete tumor response, survival, or recurrence [15]. Other authors, such as Akgun et al. in their work, have described a higher number of complete pathological responses in patients operated on more than 8 weeks since the completion of chemoradiotherapy [5]. Following other studies such as GRECCAR-6, and based on a more homogeneous distribution of our series (out of 181 patients, 87 (48.1%) were operated on in less than 8 weeks compared to 94 (51.9%) who were operated on after 8 weeks or more from completion of neoadjuvant treatment), we decided to use the reference interval of 8 weeks from the completion of neoadjuvant treatment.

The number of isolated lymph nodes after surgery and neoadjuvant treatment is controversial in rectal cancer. The number of isolated lymph nodes is known to be influenced by the effects of neoadjuvant treatment. It is known that fewer and very small lymph nodes are detected after preoperative adjuvant RT. For some authors, the detection of less than 12 lymph nodes after neoadjuvant treatment is considered a good indicator of local tumor response [16,17]. Specifically, Mechera et al. established a decrease in the number of collected lymph nodes related to the type of neoadjuvant treatment [18]. Other authors state that only in 20% of patients operated on after neoadjuvant treatment does lymphadenectomy reach 12 lymph nodes, with no relation to survival or disease-free interval [19]. Our data agree with these authors, as we have observed a higher number of positive lymph nodes in those patients with poor tumor response to neoadjuvant treatment (Mandard 3 and 4). In our series, we found that in the group of patients with a good response (Mandard 1 and 2), the mean number of lymph nodes obtained was 7.65 (1–32) with a mean metastatic involvement of 0.25 (0–6), while in the group of patients with poor response, it was 9.08 (0–31) and 1.34 (0–36).

## 5. Conclusions

The conclusion that can be drawn from this study is that patients with a higher degree of response had higher recurrence-free survival, both overall and in the form of locoregional or metastatic recurrence, as well as longer survival. However, the effect associated with the degree of tumor regression did not reach the level of statistical significance with respect to the risk of recurrence or death in multiple regression analyses. Although the degree of tumor regression is related to the evolution of tumor disease, the prognostic evolution depends more on the ypN stage and the presence of tumor deposits.

## Data Availability

The data used to support the findings of the present study are available from the corresponding author upon request.

## Supplementary Materials

The following supporting information can be downloaded at: https://www.mdpi.com/article/10.3390/cancers16101797/s1, Table S1. Clinical and demographic variables; Table S2. Pre-surgical variables related to tumor regression; Table S3. Postsurgical variables related to tumor regression.
