# Supplementary material for: Effect of Tumor Regression Grade on Survival and Disease-Free Interval in Patients Operated on for Locally Advanced Rectal Cancer
Source: Cancers (Basel). 2024 May 8;16(10):1797. doi: 10.3390/cancers16101797 (PMC11119053; doi:10.3390/cancers16101797)
Supplement: Supplementary file 1 [file cancers-16-01797-s001.zip › cancers-2960304-supplementary.pdf]

Table S1. Clinical and demographic variables.

	Total number of cases	Mandard (1-2) (n=101)	Mandard (3-5) (n=80)	p value
**Age**		62.8+/-10	62.9+/-8	0.975
**Gender**				
Male	119	68 (57,2%)	51 (42.8%)	0.639
Female	62	33 (53.2%)	29 (46.8%)	
**cT**				
T2	11	7 (63,6%)	4 (36.4%)	0.832
T3	138	77 (55.8%)	61 (44.2%)	
T4	32	17 (53.1%)	15 (46.9%)	
**cN**				0.829
N0	27	15 (55.5%)	12 (44.5%)	
N1	75	40 (55.3%)	35 (46.7%)	
N2	79	46 (58.2%)	33 (41.8%)	
**Location**				
Medium	84	48 (57.1%)	36 (42.9%)	0.426
Low	97	53 (54.6%)	44 (45.4%)	
**Stage**				
II	28	15 (53.5%)	13 (46.5%)	0.838
III	153	86 (56.2%)	67 (43.8%)	
**Stage**				
IIA	25	14 (56.0%)	11 (44.0%)	0.513
IIB	2	0	2	
IIC	2	1	0	
IIIA	7	5	2	
IIIB	115	65 (56.5%)	50 (43.5%)	
IIIC	31	16 (51.6%)	15 (48.4%)	
**Surgical Interval**				
< 8 weeks	87	47 (54.0%)	40 (46.0%)	0.656
> 8 weeks	94	54 (57.5%)	40 (42.5%)	
**NLS Index**				
> 5	26	6 (23.0%)	50 (77.0%)	0.001
< 5	155	96 (62.0%)	59 (38.0%)	
**Serum CEA levels**				
Non- elevated	125	74 (59.2%)	51 (40.8%)	0.196
Elevated	56	27 (48.2%)	29 (51.8%)	
**Serum CA 19.9 levels**				0.086
Non- elevated	155	91 (58.7%)	64 (41.3%)	
Elevated	26	10 (38.7%)	18 (69.3%)	
**Anemia**				0.398
Hb < 12 gr/dl	48	24 (50.0%)	24 (50.0%)	
Hb > 12 gr/dl	133	77 (57.9%)	56 (42.1%)	
**Albumin**				0.276
< 3,5 gr/dl	18	4 (22.2%)	14 (77.8%)	
>3,5 gr/dl	163	95 (58.3%)	68 (41.7%)	
		**Mandard (1-2) n=101)**	**Mandard (3-5) (n=80)**	**p value**
**ypT**				
ypT0	36	36 (100%)	0	0.001

ypT1	21	19 (90.5%)	2 (9.5%)	
ypT2	46	22 (47.8%)	24 (52.2%)	
ypT3	73	23 (31.5%)	50 (68.5%)	
ypT4	5	1 (20.0%)	4 (80.0%)	
**ypN**				
ypN0	131	85 (64.9%)	46 (35.1%)	0.001
ypN1	39	14 (35.9%)	25 (64.1%)	
ypN2	11	2 (18.2%)	9 (81.8%)	
**Stage**				
0	26	26 (100%)	0	0.001
I	58	42 (72.4%)	16 (27.6%)	
II	43	13 (30.2%)	30 (69.8%)	
IIIA-IIIB	48	18 (37.5%)	30 (62.5%)	
IIIC	6	2 (33.3%)	4 (66.4%)	
**Adenopathy**				
Total		7,69+/-6,08	8,9+/-5,9	0.182
Positive		0,24+/-0,78	1,34+/-2,6	0.001
**Circunferential Margin**				
Afected	11	1 (9.1%)	10 (90.1%)	0.003
Non afected	170	100 (58.8%)	70 (41.2%)	
**Grade Differentiation**				
Well/Moderate	168	98 (58.3%)	70 (41.7%)	0.019
Poorly	13	3 (23.1%)	10 (76.9%)	
**Vascular Invasion**				
Negative	165	99 (60.0%)	66 (40.0%)	0.001
Positive	16	2 (12.5%)	14 (87.5%)	
**Perineural Invasion**				
Negative	164	98 (59.8%)	66 (40.2%)	0.001
Positive	17	3 (17.6%)	14 (82.4%)	
**Tumoral Deposits**				
Negative	165	99 (60.0%)	66 (40.0%)	0.001
Positive	16	2 (12.5%)	14 (87.5%)	
**Adjuvant Chemotherapy**				
No	57	35 (61.4%)	22 (38.6%)	0.336
Yes	124	66 (53.2%)	58 (46.8%)	
**Recurrence (all types)**	41	15 (36.6%)	26 (63.4%)	0.004
Local Recurrence	5	1 (20.0%)	4 (80.0%)	0.172
Distant Metastases	38	14 (36.8%)	24 (63.2%)	0.004
Patients remaining alive	156	92 (58.9%)	64 (41,0%)	0.027

			Recurrence			Locoregional Recurrence			Distance Metastases			Overal Survival		
		Cases (%)	Cases with Recurrence	KM	p value	Cases with Recurrence	KM	p value	Cases with Metastases	KM	p value	Deceased	KM	p value
**Surgical Interval**	< 8 weeks	87 (48,1%)	17	79	0,314	1	98	0,199	17	79	0,595	9	88	0,206
	> 8 weeks	94 (51,9%)	24	73		4	95		21	76		16	81	
**Location**	Medium	84 (46,4%)	24	69	0,076	1	98	0,275	23	70	0,050	16	76	0,032
	Low	97 (53,6%)	17	81		4	95		15	83		8	90	
**Clinical Stage**	Stage II	28 (15,5%)	10	63	0,076	2	92	0,139	9	66	0,123	4	84	0,925
	Stage III	153 (84,5%)	31	78		3	97		29	80		20	85	
**Clinical Stage**	Iia	25(13,8%)	9	62	0,016	2	92		8	65	0,012	4	82	0,661
	IIb	2 (1,1%)	0	100		0	100		0	100		0	100	
	IIc	1 (0,6%)	1	100		0	100		1	0		0	100	
	IIIa	7(3,9%)	3	57		0	100		3	57		2	68	
	IIIb	115 (63,5%)	19	82		3	96		17	84		13	87	
	IIIc	31 (17,1%)	9	68		0	100		9	6		6	78	
**Gender**	Male	119(657%)	26	76	0,779	4	96	0,483	24	78	0,764	15	86	0,75
	Female	62(34,3%)	15	75		1	98		14	76		9	84	
**cT**	T2	11 (6,1%)	4	63		0	100	0,467	4	63	0,194	2	80	0,583
	T3	138 (76,2%)	28	78		5	95		25	81		17	86	
	T4	32 (17,7%)	9	69		0	100		9	69		6	79	
**cN**	N0	27 (14,9%)	10	61	0,129	2	92	0,267	9	64	0,197	4	84	0,98
	N1	75 (41,4%)	17	76		2	96		16	77		10	85	
	N2	79 (43,6%)	14	81		1	98		13	82		11	84	
**CEA**	Non -elevated	125 (69%)	29	76	0,969	4	96	0,638	27	77	0,903	19	83	0,581
	Elevated	56 (31%)	12	76		1	97		11	7		6	87	
**CA 19.9**	Non -elevated	155 (85,6%)	32	78	0,101	5	96	0,387	29	80	0,057	19	86	0,305
	Elevated	26 (14,4%)	9	62		0	100		9	62		6	78	
**Hemoglobin**	Hb < 12 gr/dl	48 (26,5%)	12	72	0,576	1	97	0,776	11	74	0,633	9	78	0,222
	Hb > 12 gr /dl	133 (73,5%)	29	77		4	96		27	78		16	87	
**Albumin**	< 3,5 gr/dl	15 (8,3%)	3	75	0,988	1	90	0,165	2	82	0,606	1	91	0,565
	> 3,5 gr/dl	159 (87,8%)	35	77		3	97		33	78		22	84	
**NLS**	> 5	25 (13,8%)	9	54	0,048	2	90	0,063	8	59	0,092	5	73	0,195
	< 5	155 (85,6%)	32	78		3	97		30	80		20	86	
**NLS**	< 3	110 (60,8%)	23	78	0,408	3	96	0,898	21	80	0,369	16	84	0,918
	> 3	70 (38,7%)	18	71		2	96		17	72		9	85	
**ILL**	> 25,7%	98 (54,1%)	23	74	0,803	2	97	0,522	22	75	0,626	13	85	0,838
	< 25;7%	82 (45,3%)	18	77		3	96		16	80		12	84	

Table S2. Pre-surgical variables related to tumor regression.

Table S3. Postsurgical variables related to tumor regression.

			Recurrence			Locoregional Recurrence			Distance Metastases			Overal Survival		
		Cases (%)	Cases with Recurrence	KM	p value	Cases with Recurrence	KM	p value	Cases with Metastases	KM	p value	Deceased	KM	p value
ypT	ypT0	36 (19,8%)	6	82	< 0,001	1	97	0,823	5	85	0,001	5	85	0,002
	ypT1	21 (11,6%)	1	95		0	100		1	95		1	95	
	ypT2	46 (25,4%)	6	86		1	97		6	86		3	93	
	ypT3	73 (40,3%)	25	62		3	94		23	65		13	79	
	ypT4	5 (2,8%)	3	40		0	100		3	40		3	40	
ypN	ypN0	131 (72,4%)	19	85	< 0,001	4	96	0,136	16	87	< 0,001	11	91	< 0,001
	ypN1	39 (21,5%)	16	54		0	100		16	54		9	71	
	ypN2	11 (6,1%)	6	34		1	85		6	34		5	45	
Stage	0	26 (14,4%)	4	84	< 0,001	1	96	0,326	3	88	< 0,001	3	87	< 0,001
	I	58 (32%)	4	93		0	100		4	93		3	94	
	II	43 (23,8%)	10	75		3	92		8	80		4	90	
	IIIa-IIIb	48 (26,5%)	19	55		1	97		19	55		11	71	
	IIIc	6 (3,3%)	4	25		0	100		4	25		4	0	
Mandard	1	32 (17,6%)	4	87	0,011	1	96	0,148	3	90	0,023	3	87	0,107
	2	69 (38,1%)	11	83		0	100		11	83		6	90	
	3	67 (37%)	21	66		3	92		20	68		14	77	
	4	13 (7,2%)	5	51		1	90		4	61		2	77	
	5	0	0	0		0						0		
Grouped Mandard	1-2	101 (55,8%)	15	84	0,002	1	99	0,032	14	85	0,003	9	90	0,014
	3-4-5	80 (44,2%)	26	64		4	91		24	67		16	77	
Circumferential Margin	Negative	170 (93,9%)	35	78	< 0,001	5	96	0,651	32	80	< 0,001	20	87	< 0,001
	Positive	11 (6,1%)	6	37		0	100		6	37		5	50	
Grade Differentiation	Well/Moderate	168 (92,8%)	36	75	0,127	5	96	0,555	33	79	0,088	21	86	0,046
	Poorly	13 (7,2%)	5	58		0	100		5	58		4	67	
Vascular Invasion	Negative	165 (91,2%)	31	80	< 0,001	5	96	0,522	28	82	< 0,001	20	86	0,009
	Positive	16 (8,8%)	10	31		0	100		10	31		5	65	
Perineural Invasion	Negative	164 (90,6%)	31	80	< 0,001	5	96	0,523	28	82	< 0,001	20	86	0,004
	Positive	17 (9,4%)	10	31		0	100		10	31		5	64	
Tumoral Deposits	Negative	165 (91,2%)	31	80	0,001	4	97	0,01	28	81	< 0,001	19	87	< 0,001
	Positive	16 (8,9%)	10	36		1	76		10	36		6	57	
Adjuvant Chemotherapy	No	57 (31,5%)	11	79	0,428	2	96	0,699	9	83	0,228	4	92	0,076
	Yes	124 (68,5%)	30	74		3	97		29	75		21	81	
